# Sex-Specific Association Between Iron Status and the Predicted 10-Year Risk for Atherosclerotic Cardiovascular Disease in Hypertensive Patients

**DOI:** 10.1007/s12011-021-03060-y

**Published:** 2022-01-24

**Authors:** Juan Zhou, Rui Zhao, Dongxia Wang, Qin Gao, Dan Zhao, Binfa Ouyang, Liping Hao, Xiaolin Peng

**Affiliations:** 1 Shenzhen Nanshan Center for Chronic Disease Control, Shenzhen, 518051 China; 2 Department of Nutrition and Food Hygiene, Hubei Key Laboratory of Food Nutrition and Safety, and Ministry of Education Key Laboratory of Environment and Health, School of Public Health, Tongji Medical College, Huazhong University of Science and Technology, Wuhan, 430030 China; 3 Department of Public Health, Jining Medical University, Jining, 272067 China; 4 Department of Oncology, Injury Prevention and Nutrition, Shenzhen Nanshan Center for Chronic Disease Control, 7 Huaming Road, Shenzhen, 518051 China

**Keywords:** Iron, Ferritin, Haemoglobin, Atherosclerotic cardiovascular disease, Hypertension, Sex-specific

## Abstract

Serum ferritin (SF) and haemoglobin (Hb) are widely used in clinical practice to assess iron status. Studies exploring the relationship of SF and Hb with atherosclerotic cardiovascular disease (ASCVD) risk have yielded conflicting results, and some indicated sex specificity. Hypertensive patients have abnormal iron status. However, research on patients with hypertension is limited. We aim to investigate the sex-specific links of SF and Hb with the predicted 10-year ASCVD risk in hypertensive patients. This cross-sectional study included 718 hypertensive men and 708 hypertensive women. The predicted 10-year ASCVD risk was calculated based on the China-PAR equation. The dose–response curves were illustrated by fitting linear and quadratic models. In hypertensive men, the iron status fits for a quadratic model for ASCVD risk, showing a U-shape. After adjusting for potential confounding factors, the regression coefficients and 95% confidence intervals (95% CI) across tertile of SF were 0.0 (reference), − 0.99 (− 1.65, − 0.33) and − 0.22 (− 0.88, 0.44), and of Hb were 0.0 (reference), − 0.74 (− 1.41, − 0.08) and − 0.77 (− 1.46, − 0.08). In hypertensive women, iron status was linearly and positively associated with ASCVD risk. Per one unit increment of log-transformed SF as well as Hb was associated with a 1.22 (95% CI: 0.97, 1.48) and 0.04 (95% CI: 0.02, 0.07) increased in ASCVD risk score, respectively. A significant interaction between iron status and inflammation on ASCVD risk was observed in hypertensive women. SF and Hb showed a U-shape with ASCVD risk in hypertensive men; however, a positive linear relationship was observed in hypertensive women.

**Supplementary Information:**

The online version contains supplementary material available at 10.1007/s12011-021-03060-y.

## Introduction

Iron is an essential trace mineral that plays an irreplaceable role in the normal physiological processes of the body [1]. Iron deficiency (ID) is the leading cause of anaemia and is characterised by the insufficient synthesis of haemoglobin (Hb) [2]. Contrarily, free iron is highly reactive. To reduce oxidative damage, the iron that exceeds metabolic requirements is stored intracellularly as ferritin [3]. Consequently, the amount of iron deposited is closely reflected by the concentration of serum ferritin (SF). Clinically, Hb and SF are widely used as indicators of iron status [4].

The association between iron status and cardiovascular disease has been studied extensively since Sullivan proposed the iron-heart hypothesis [5], which suggested that increased iron deposits contribute to the risk of cardiovascular disease. Although several subsequent prospective studies backed up this theory, showing that elevated SF concentrations were linked to an increased risk of carotid atherosclerosis [6] and myocardial infarction (MI) [7], a huge body of epidemiological research has not confirmed this connection [8–12]. Furthermore, some surveys indicated that low iron status may also be an independent risk factor for coronary artery disease and stroke [13, 14], and with sex specificity [15]. For example, a large prospective cohort study in community-living adults found the U-shape association between Hb with stroke incidents only in women [15]. The sex-specific association between iron status and cardiovascular disease risk remains to be further explored.

Hypertension, a well-known contributor to atherosclerotic cardiovascular disease (ASCVD), is suggested to be related to abnormal iron status [16]. When compared to the non-hypertensive individuals, the SF and Hb levels are markedly higher in hypertensive patients [16]. Indeed, the perturbation of iron status is more pronounced when aortic stiffness is increased in hypertensive patients [17]. The previous study has noted that excess iron affects the insulin resistance state and sympathetic neural function in hypertensive patients [18], and both of which are highly related to ASCVD [19, 20]. Thus, the abnormal iron status may be a potential key factor in the development of ASCVD in hypertensive patients. However, information on the relationship between iron status and ASCVD risk in individuals with hypertension is extremely limited.

Clinically, the composite risk score is the standard approach for predicting future ASCVD risk, determining the need for further testing and initiating therapeutic intervention [21]. The predicted 10-year ASCVD risk, derived from the Prediction model for ASCVD risk in China (China-PAR equations), represents the preclinical stage of comprehensive ASCVD risk and can guide public health and clinical practice [22]. Few studies had looked at the relationship between iron status and the predicted 10-year ASCVD risk.

In this context, we aimed to explore the sex-specific association of iron status with the predicted 10-year ASCVD risk in a cross-sectional study comprising 718 hypertensive men and 708 hypertensive women from Shenzhen, China.

## Methods

### Study Population

In a cross-sectional study, we randomly recruited 1992 hypertensive patients at 53 community health service centres in the Nanshan District, Shenzhen, China, during 2017. These patients had been diagnosed with hypertension by the clinical physicians prior to the survey. As the China-PAR model was developed in a population aged 35–74 years and without a history of ASCVD [22], we excluded individuals aged < 35 years (*n* = 23) and aged > 74 years (*n* = 170), as well as those patients with pre-existing clinical cardiovascular disease, including stroke, coronary heart disease and heart failure (*n* = 228). Individuals with self-reported kidney and liver disorder (*n* = 21), cancer (*n* = 14) and with an estimated glomerular filtration rate ≤ 60 mL/min/1.73 m^2^ (*n* = 61) and alanine aminotransferase levels > 3 times of the upper normal limit (*n* = 5) were also excluded, as these patients possibility had abnormal iron status. After excluding those with missing data on ASCVD risk score (*n* = 44), 1426 patients (718 men and 708 women) were eventually enrolled in this study (Fig. S1).

The survey was approved by the Ethics Committee of the Shenzhen Nanshan Centre for Chronic Disease Control and was performed following the Declaration of Helsinki and its amendments. Written informed consent was received from all included patients.

### Data and Biospecimen Collection

The questionnaire, anthropometric measurements and blood sample collection were all taken on the same day. Trained research staff obtain demographic information, personal and family medical history, personal medication history and lifestyle information through a standardised questionnaire. Anthropometric data, including height, weight and waist circumference (WC), were acquired by direct measurements. Body mass index (BMI) was calculated by dividing weight in kilograms by the squared value of height in metres. Blood pressure (BP) was measured twice in a sitting position with an automated BP instrument (Omron-705CP; Omron Corp., Tokyo, Japan).

Morning blood samples were collected for each participant who fasted for more than 10 h. Total cholesterol (TC), triglycerides (TG), high-density lipoprotein cholesterol (HDL-C), low-density lipoprotein cholesterol (LDL-C), fasting blood glucose (FBG), high-sensitivity C-reactive protein (hs-CRP), alanine aminotransferase and creatinine were determined by the automatic analyser (Hitachi 7080, Tokyo, Japan). SF was determined by Abbotti2000SR automatic chemiluminescence immunoassay. The concentrations of Hb were acquired on an automated Mindray BC-5800 analyser (Mindray, Shenzhen, China).

### Predicted 10-Year Risk for ASCVD

The 10-year ASCVD risk prediction is a useful tool to assess the risk of developing a first ASCVD event, including nonfatal MI, coronary heart disease death, fatal or nonfatal stroke, within 10 years in people free of ASCVD [23]. The China-PAR prediction equation, derived from sex-specific Cox proportional hazard models, has been shown to outperform the Pooled Cohort Equations in predicting 10-year ASCVD risk in the Chinese population [22]. The China-PAR model includes the following variables: sex, age, geographic region (northern/southern), urbanization (urban/rural), WC, TC, HDL-C, systolic BP (SBP), diastolic BP (DBP), treatment of hypertension (yes/no), diabetes mellitus (yes/no), current smoker (yes/no), family history of ASCVD (yes/no) and the interaction terms for age with risk factors that met predetermined statistical criteria. All continuous variables included in the equations were converted to natural logarithms and the categorical variables were expressed as 1 or 0. The equation form is expressed as:

$$\text{The predicted 10}-\text{year risk of ASCVD} \, \left(\%\right)=1-{\text{S}}_{10}{{\text{e}}}^{\left(\mathrm{IndX'B}-\mathrm{MeanX'B}\right)}$$

For the equation, the *S*_10_ is the baseline survival rate for ASCVD at 10 years. IndX’B is the individual sum, which is defined as the sum of “coefficient × value”. MeanX’B is the overall mean “coefficient × value”. The sex-specific values of *S*_10_ and the coefficient, as well as the mean sum for the equation, were described in detail elsewhere [22].

### Ascertainment of Components of China-PAR and Covariates

Marital status was divided into married and unmarried. Divorced, widowed and cohabiting were included as unmarried. Education level was classified into primary school or below, high school and college or above. The definition of the current smoker and diabetes mellitus has been previously defined [24]. Individuals who had an alcoholic drink at least once a week for more than 6 months were categorised as current drinkers. Physical activity was described as exercising at least three a week for at least 20 min each time over the last 6 months. Treatment of hypertension was identified as self-reported use of a prescription drug for hypertension during the past 2 weeks, control as SBP < 140 mm Hg and DBP < 90 mm Hg. Based on the Chinese Guidelines on Prevention and Treatment of Dyslipidemia in Adults, the presence of abnormal serum lipid concentrations (TC ≥ 6.2 mmo/L or TG ≥ 2.3 mmo/L or LDL-C ≥ 4.1 mmo/L or HDL-C < 1.0 mmo/L), or the use of lipid-lowering drugs within 2 weeks at the time of the survey were used to define dyslipidemia [25]. Family history of ASCVD was described as having at least a parent or a sibling with a stroke or MI [22]. Anaemia was defined as a Hb level below 130 g/L for men and below 120 g/L for women. ID was described as SF less than 30 ng/mL [26].

### Statistical Analysis

In this study, SF and Hb were used to assess iron status. Relation between SF and Hb was tested using the Pearson test. Circulating ferritin concentration was right-skewed and therefore logarithmically transformed to approximate a normal distribution.

For descriptive purposes, we divided participants of each sex into three groups according to tertiles of iron status. We estimated means (SDs) and medians (interquartile ranges [IQRs]) for continuous variables and counts (percentages) for categorical variables. Linear regression for continuous variables (with the median concentration of each tertile as the variable used in the model) and *χ*^2^ with the linear-by-linear association test for categorical variables was used for linear trend tests across tertiles.

The generalised linear models were conducted to quantify the correlation of iron status (as a categorical variable) with the predicted 10-year ASCVD risk. As the results suggest a nonlinear quadratic relationship between iron status and 10-year ASCVD risk score in men (both *P* for quadratic trend < 0.05). Consequently, we added a quadratic term for iron status in the multivariable-adjusted linear regression model for the predicted probability of future ASCVD risk and graphically presented the dose–response curve. If there is no evidence for a quadratic trend, we calculated the change in ASCVD risk score for each one-unit increase in the iron status with the use of linear regression models.

To control for potentially influential factors, we adjusted for marital status, education level, current drinker, physical activity, BMI and hs-CRP. The age and other variables were not included in the adjusted models as it is a component of the China-PAR equation. Adjusting for variables included in the China-PAR equation may result in over-adjustment. Inflammation affects levels of SF and Hb irrespective of iron status [27] and increases the risk of ASCVD [28]. To explore if the association between iron status and ASCVD risk was consistent across hs-CRP levels, we performed a subgroup analysis testing for interaction (hs-CRP × iron status) stratifying by median hs-CRP (1.3 mg/L).

All analyses were generated using R statistical software version 4.0.4. *P*-values below 0.05 indicate statistical significance.

## Results

### Characteristics of Participants Stratified by Sex

As presented in Table 1, compared to women, men had a higher proportion of married, highly educated, current smoker, current drinker and dyslipidaemia, and higher BMI, WC, SF, Hb and 10-year ASCVD score (all *P*-value < 0.05). Besides, men were younger and had a lower prevalence of ID and anaemia than women (all *P*-value < 0.05). There were no statistical differences between the sex in the distribution of physical activity (yes/no), diabetes mellitus (yes/no), family history of ASCVD (yes/no), treatment of hypertension (yes/no), control of hypertension (yes/no) and hs-CRP (all *P*-value > 0.05).

**Table 1:** Characteristics of the hypertensive population stratified by sex (Data are expressed as the means ± SDs for normally distributed variables or as the median [interquartile range] for non-normally distributed variables, or counts (percentages) for categorical variables)

Characteristics	Total (*n* = 1426)	Men (*n* = 718)	Women (*n* = 708)	*P*-value^a^
Age (years)	56.15 ± 9.64	54.37 ± 9.88	57.96 ± 9.05	< 0.001
Marital status, *n* (%)				
Married	1347 (94.5)	695 (96.8)	652 (92.1)	< 0.001
Unmarried	79 (5.5)	23 (3.2)	56 (7.9)	
Education level, *n* (%)				
Primary school or below	270 (18.9)	61 (8.5)	209 (29.5)	< 0.001
High school	800 (56.1)	415 (57.8)	385 (54.4)	
College or above	356 (25.0)	242 (33.7)	114 (16.1)	
Current smoker, *n* (%)				
No	1223 (85.8)	519 (72.3)	704 (99.4)	< 0.001
Yes	203 (14.2)	199 (27.7)	4 (0.6)	
Current drinker, *n* (%)				
No	1009 (70.8)	382 (53.2)	627 (88.6)	< 0.001
Yes	417 (29.2)	336 (46.8)	81 (11.4)	
Physical activity, *n* (%)				
No	515 (36.1)	254 (35.4)	261 (36.9)	0.596
Yes	911 (63.9)	464 (64.6)	447 (63.1)	
Diabetes mellitus, *n* (%)				
No	1131 (79.3)	578 (80.5)	553 (78.1)	0.293
Yes	295 (20.7)	140 (19.5)	155 (21.9)	
Dyslipidaemia, *n* (%)				< 0.001
No	743 (52.1)	334 (46.5)	409 (57.8)	
Yes	683 (47.9)	384 (53.5)	299 (42.2)	
Family history of ASCVD, *n* (%)				
No	1126 (79.0)	577 (80.4)	549 (77.5)	0.215
Yes	300 (21.0)	141 (19.6)	159 (22.5)	
Treatment of hypertension, *n* (%)				
No	379 (26.6)	188 (26.2)	191 (27.0)	0.780
Yes	1047 (73.4)	530 (73.8)	517 (73.0)	
Control of hypertension, *n* (%)				0.092
No	562 (39.4)	299 (41.6)	263 (37.1)	
Yes	864 (60.6)	419 (58.4)	445 (62.9)	
ID, *n* (%)				< 0.001
No	1323 (92.8)	706 (98.3)	617 (87.1)	
Yes	103 (7.2)	12 (1.7)	91 (12.9)	
Anaemia, *n* (%)				< 0.001
No	1232 (86.4)	647 (90.1)	585 (82.6)	
Yes	194 (13.6)	71 (9.9)	123 (17.4)	
BMI (kg/m^2^)	25.31 ± 3.26	25.88 ± 3.13	24.74 ± 3.28	< 0.001
WC (cm)	87.84 ± 9.40	91.12 ± 8.96	84.52 ± 8.65	< 0.001
hs-CRP (mg/L)	1.30 [0.70, 2.40]	1.30 [0.70, 2.40]	1.35 [0.70, 2.50]	0.681
SF (ng/mL)	163.00 [92.37, 254.85]	215.12 [145.53, 324.59]	110.58 [59.09, 180.37]	< 0.001
Hb (g/L)	136.98 ± 13.86	145.18 ± 11.85	128.67 ± 10.36	< 0.001
ASCVD risk score (%)	5.30 [3.20, 7.80]	5.70 [3.50, 8.20]	4.90 [3.08, 7.50]	< 0.001

*ASCVD*, atherosclerotic cardiovascular disease; *BMI*, body mass index; *Hb*, haemoglobin; *hs****-****CRP*, high sensitivity C-reactive protein; *ID*, iron deficiency; *SF*, serum ferritin; *WC*, waist circumference

^a^*P*-value was derived from Student’s *t*-test and Mann–Whitney *U* tests for continuous variables and chi-square tests for categorical variables

**Fig. 1:**
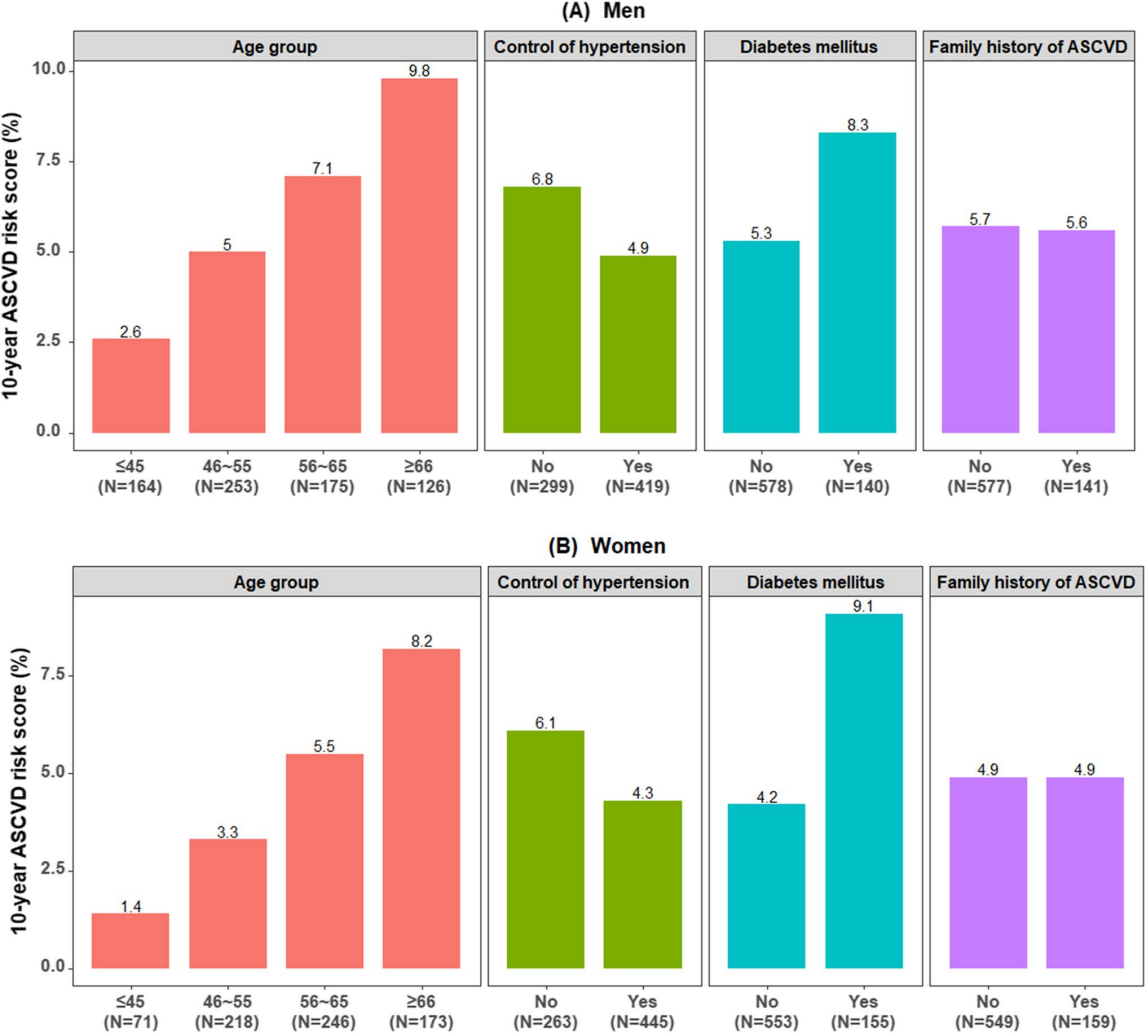
**A**,** B** Distribution of the estimated 10-year ASCVD risk score in hypertensive patients, stratified by age group, control of hypertension, diabetes mellitus and family history of ASCVD. The *Y*-axis represents the 10-year ASCVD risk score. ASCVD, atherosclerotic cardiovascular disease

Figure 1 shows the distribution of the estimated 10-year ASCVD risk score in the different subgroups. It was obvious that the predicted 10-year ASCVD risk was higher in the elderly, those with poorly controlled hypertension and those with diabetes (*P*-value < 0.05).

### Characteristics of Participants According to the Tertiles of Iron Status

As shown in Table 2, men with higher SF levels had greater TG and hs-CRP and tended to be younger and current drinkers (all *P* for trend < 0.05). However, such a linear trend was not observed for the 10-year ASCVD risk score (*P* for trend > 0.05). Additionally, the men in the highest tertile of Hb had greater BMI, TG and LDL-C, lower ASCVD risk score and tended to be younger (all *P* for trend < 0.05). As presented in Table 3, women with higher SF concentration had greater LDL-C, hs-CRP and 10-year ASCVD risk scores, and were more likely to be elder, unmarried and physically active (all *P* for trend < 0.05). Also, the women in the highest tertile of Hb had greater BMI, LDL-C, hs-CRP and 10-year ASCVD risk score (all *P* for trend < 0.05). The distribution of the variables included in the China-PAR model stratified by tertiles of iron status in men and women is presented in Table S1 and Table S2, respectively.

**Table 2:** Characteristics of the hypertensive population according to tertiles of iron status in men (*n* = 718) (Data are expressed as the means ± SDs for normally distributed variables or as the median [interquartile range] for non-normally distributed variables, or counts (percentages) for categorical variables)

Characteristics	Tertiles of SF				Tertiles of Hb			
	T1	T2	T3	*P* for trend^a^	T1	T2	T3	*P* for trend^a^
Age (years)	57.09 ± 9.86	53.18 ± 9.91	52.89 ± 9.34	< 0.001	57.77 ± 10.24	54.01 ± 9.38	51.60 ± 9.15	< 0.001
Marital status, *n* (%)				0.602				0.509
Married	229 (96.6)	235 (96.3)	231 (97.5)		218 (98.6)	246 (94.6)	231 (97.5)	
Unmarried	8 (3.4)	9 (3.7)	6 (2.5)		3 (1.4)	14 (5.4)	6 (2.5)	
Education level, *n* (%)				0.004				0.829
Primary school or below	29 (12.2)	23 (9.4)	9 (3.8)		20 (9.0)	21 (8.1)	20 (8.4)	
High school	127 (53.6)	135 (55.3)	153 (64.6)		127 (57.5)	149 (57.3)	139 (58.6)	
College or above	81 (34.2)	86 (35.2)	75 (31.6)		74 (33.5)	90 (34.6)	78 (32.9)	
Current drinker, *n* (%)				0.008				0.001
No	140 (59.1)	131 (53.7)	111 (46.8)		141 (63.8)	133 (51.2)	108 (45.6)	
Yes	97 (40.9)	113 (46.3)	126 (53.2)		80 (36.2)	127 (48.8)	129 (54.4)	
Physical activity, *n* (%)				0.055				0.017
No	67 (28.3)	100 (41.0)	87 (36.7)		65 (29.4)	94 (36.2)	95 (40.1)	
Yes	170 (71.7)	144 (59.0)	150 (63.3)		156 (70.6)	166 (63.8)	142 (59.9)	
BMI (kg/m^2^)	25.40 ± 2.95	26.16 ± 3.23	26.05 ± 3.17	0.050	24.93 ± 2.99	26.16 ± 3.21	26.44 ± 2.98	< 0.001
TG (mmol/L)	1.43 [0.95, 2.01]	1.66 [1.14, 2.36]	1.84 [1.46, 2.70]	< 0.001	1.41 [0.96, 1.96]	1.73 [1.20, 2.33]	1.81 [1.26, 2.56]	< 0.001
LDL-C (mmol/L)	3.20 ± 0.74	3.25 ± 0.75	3.17 ± 0.74	0.626	3.07 ± 0.65	3.24 ± 0.81	3.30 ± 0.74	0.002
hs-CRP (mg/L)	1.20 [0.70, 2.10]	1.30 [0.70, 2.30]	1.40 [0.80, 2.90]	0.006	1.30 [0.70, 2.36]	1.40 [0.80, 2.52]	1.20 [0.70, 2.20]	0.062
SF (ng/mL)	117.01 [85.57, 144.30]	215.12 [190.69, 243.35]	386.62 [325.71, 484.93]	< 0.001	210.33 [128.45, 327.44]	207.32 [140.01, 312.45]	234.09 [165.02, 329.58]	0.010
Hb (g/L)	143.59 ± 11.96	146.34 ± 11.47	145.57 ± 12.01	0.133	131.65 ± 6.91	145.14 ± 3.09	157.84 ± 6.29	< 0.001
ASCVD risk score (%)	6.50 [4.40, 9.00]	4.95 [2.80, 7.70]	5.60 [3.50, 8.00]	0.242	6.70 [4.30, 9.40]	5.45 [3.58, 7.90]	5.20 [3.00, 7.60]	0.005

*ASCVD*, atherosclerotic cardiovascular disease; *BMI*, body mass index; *Hb*, haemoglobin; *hs****-****CRP*, high sensitivity C-reactive protein; *LDL-C*, low-density lipoprotein cholesterol; *SF*, serum ferritin; *T*, tertile; *TG*, triglyceride

^a^Linear trends were tested using linear regression for continuous variables (with the median levels of SF or Hb as a continuous variable included in the regression models), and *χ*^2^ with linear-by-linear association test for categorical variables

**Table 3:** Characteristics of the hypertensive population according to tertiles of iron status in women (*n* = 708) (Data are expressed as the means ± SDs for normally distributed variables or as the median [interquartile range] for non-normally distributed variables, or counts (percentages) for categorical variables)

Characteristics	Tertiles of SF				Tertiles of Hb			
	T1	T2	T3	*P* for trend^a^	T1	T2	T3	*P* for trend^a^
Age (years)	52.32 ± 9.37	59.57 ± 7.85	61.94 ± 6.85	< 0.001	57.47 ± 9.57	58.00 ± 8.72	58.33 ± 8.89	0.305
Marital status, *n* (%)				0.004				0.214
Married	225 (96.2)	219 (91.2)	208 (88.9)		198 (90.4)	210 (92.1)	244 (93.5)	
Unmarried	9 (3.8)	21 (8.8)	26 (11.1)		21 (9.6)	18 (7.9)	17 (6.5)	
Education level, *n* (%)				0.317				0.527
Primary school or below	72 (30.8)	69 (28.7)	68 (29.1)		66 (30.1)	66 (28.9)	77 (29.5)	
High school	115 (49.1)	133 (55.4)	137 (58.5)		110 (50.2)	126 (55.3)	149 (57.1)	
College or above	47 (20.1)	38 (15.8)	29 (12.4)		43 (19.6)	36 (15.8)	35 (13.4)	
Current drinker, *n* (%)				0.884				.0.646
No	207 ( 88.5)	212 (88.3)	208 (88.9)		194 (88.6)	205 (89.9)	228 (87.4)	
Yes	27 ( 11.5)	28 (11.7)	26 (11.1)		25 (11.4)	23 (10.1)	33 (12.6)	
Physical activity, *n* (%)				0.017				0.097
No	102 (43.6)	82 (34.2)	77 (32.9)		89 (40.6)	85 (37.3)	87 (33.3)	
Yes	132 (56.4)	158 (65.8)	157 (67.1)		130 (59.4)	143 (62.7)	174 (66.7)	
BMI (kg/m^2^)	24.63 ± 3.43	24.63 ± 3.04	24.96 ± 3.38	0.238	24.50 ± 3.55	24.31 ± 3.07	25.31 ± 3.16	0.004
TG (mmol/L)	1.42 [0.91, 1.92]	1.41 [1.05, 1.87]	1.51 [1.21, 2.07]	0.148	1.41 [0.97, 1.90]	1.41 [1.08, 1.91]	1.50 [1.12, 2.09]	0.165
LDL-C (mmol/L)	3.19 ± 0.84	3.32 ± 0.73	3.40 ± 0.96	0.008	3.13 ± 0.75	3.25 ± 0.80	3.50 ± 0.93	< 0.001
hs-CRP (mg/L)	1.10 [0.70, 2.10]	1.40 [0.70, 2.60]	1.60 [0.80, 2.70]	< 0.001	1.10 [0.70, 2.43]	1.30 [0.70, 2.36]	1.60 [0.90, 2.60]	0.015
SF (ng/mL)	41.98 [21.42, 58.52]	110.58 [94.13, 129.91]	220.13 [181.73, 288.01]	< 0.001	105.78 [33.01, 170.93]	106.08 [62.74, 161.12]	128.79 [79.83, 204.44]	< 0.001
Hb (g/L)	125.85 ± 11.26	129.46 ± 8.88	130.69 ± 10.26	< 0.001	116.85 ± 7.17	128.51 ± 2.31	138.73 ± 4.97	< 0.001
ASCVD risk score (%)	3.60 [1.83, 5.50]	5.20 [3.30, 7.43]	6.10 [4.20, 8.90]	< 0.001	4.60 [2.75, 6.80]	4.70 [3.10, 7.50]	5.30 [3.30, 7.90]	0.020

*ASCVD*, atherosclerotic cardiovascular disease; *BMI*, body mass index; *Hb*, haemoglobin; *hs****-****CRP*, high sensitivity C-reactive protein; *LDL-C*, low-density lipoprotein cholesterol; *SF*, serum ferritin; *T*, tertile; *TG*, triglyceride

^a^Linear trends were tested using linear regression for continuous variables (with the median levels of SF or Hb as a continuous variable included in the regression models), and *χ*^2^ with linear-by-linear association test for categorical variables

Figure S2 illustrates the frequency histogram of log-transformed SF, as well as the Hb concentration. Pearson *r*-test indicated that log-transformed SF was positively associated with Hb (men:* r* = 0.093, *P* = 0.013; women:* r* = 0.304, *P* < 0.001; Fig. S3).

### Association Between Iron Status and 10-Year ASCVD Risk Score

**Fig. 2:**
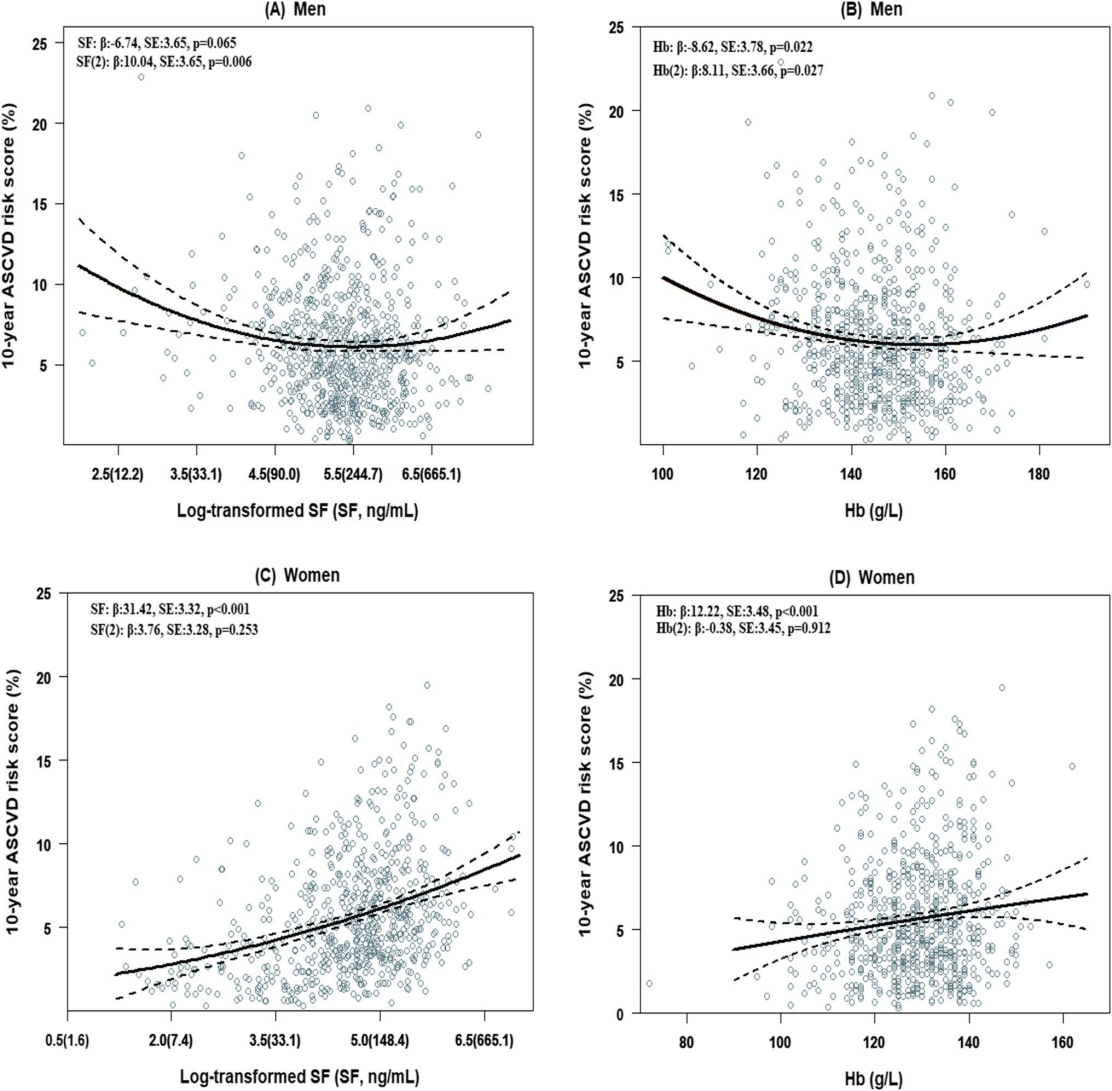
**A**–**D** Predicted dose–response curves of 10-year ASCVD risk score by linear regression model in quadratic for iron status. The black solid lines correspond to the predicted line, and the black dotted lines corresponded to the confidence intervals. Models adjusted for marital status, education level, current drinker, physical activity, BMI and hs-CRP. ASCVD, atherosclerotic cardiovascular disease; Hb, haemoglobin; SF, serum ferritin

Table 4 presents the association between iron status and 10-year ASCVD risk score. For men, relative to the lowest tertile, the adjusted regression coefficients (*β*) and 95% confidence interval (95% CI) of the second and third tertile of SF were − 0.99 (95% CI: − 1.65, − 0.33) and − 0.22 (95% CI: − 0.88, 0.44), and for Hb were − 0.74 (95% CI: − 1.41, − 0.08) and − 0.77 (95% CI: − 1.46, − 0.08), respectively. The dose–response curve in men seems to be U-shape (Fig. 2A and B), as the quadratic SF term, as well as quadratic Hb term, was significantly related to the 10-year ASCVD risk score (SF (2): *β*: 10.04, SE: 3.65, *P*-value = 0.006; Hb (2): *β*: 8.11, SE: 3.66, *P*-value = 0.027). Contrary to men, the 10-year ASCVD risk score for women increased in a dose-dependent manner with increasing SF and Hb (Table 4), and the quadratic models were not fit for women (Fig. 1C and D). In the continuous analyses, per one unit increase of log-transformed SF as well as Hb was associated with a 1.22 (95% CI: 0.97, 1.48) and 0.04 (95% CI: 0.02, 0.07) increased in ASCVD risk score, respectively (Table 4).

**Table 4:** Association between iron status and 10-year ASCVD risk score in the hypertensive population

	Tertiles of SF (ng/mL)					Tertiles of Hb (g/L)				
	T1	T2	T3	*P* for trend^a^	Per one log-transformed SF	T1	T2	T3	*P* for trend^a^	Per one Hb
Men										
Range	< 168.6	168.6 ~ 279.5	> 279.5			< 140.0	140.9 ~ 150.4	> 150.4		
Crude model	0.0 (Reference)	− 1.21 (− 1.89, − 0.54)^***^	− 0.44 (− 1.12, 0.23)	0.460	-	0.0 (Reference)	− 0.83 (− 1.50, − 0.15)^*^	− 1.00 (− 1.69, − 0.30)^**^	0.005	-
Adjusted model^b^	0.0 (Reference)	− 0.99 (− 1.65, − 0.33)^**^	− 0.22 (− 0.88, 0.44)	0.896	-	0.0 (Reference)	− 0.74 (− 1.41, − 0.08)^*^	− 0.77 (− 1.46, − 0.08)^*^	0.033	-
Women										
Range	< 78.5	78.5 ~ 153.8	> 153.8			< 125.0	125.0 ~ 133.0	> 133.0		
Crude model	0.0 (Reference)	1.56 (0.94, 2.19)^***^	2.97 (2.35, 3.59)^***^	< 0.001	1.33 (1.06, 1.59)^***^	0.0 (Reference)	0.35 (− 0.33, 1.02)	1.00 (0.35, 1.65)^**^	0.002	0.05 (0.03, 0.08)^***^
Adjusted model^b^	0.0 (Reference)	1.44 (0.85, 2.04)^***^	2.71 (2.11, 3.31)^***^	< 0.001	1.22 (0.97, 1.48)^***^	0.0 (Reference)	0.38 (− 0.26, 1.02)	0.75 (0.13, 1.38)^*^	0.018	0.04 (0.02, 0.07)^***^

*ASCVD*, atherosclerotic cardiovascular disease; *Hb*, haemoglobin; *hs****-****CRP*, high sensitivity C-reactive protein; *SF*, serum ferritin; *T*, tertile

^a^*P* for trend were performed by assigning each participant the median value of including the tertile of iron status and treating it as a continuous variable

^b^Adjusted model was adjusted for marital status, education level, current drinker, physical activity, BMI and hs-CRP

^*^P < 0.05; ^**^P < 0.01; ^***^P < 0.001

### Interaction Between Iron Status and hs-CRP in Relation to 10-Year ASCVD Risk Score

Participants were divided into two groups using the median hs-CRP as the cut-off point to explore the interaction between iron status and hs-CRP in relation to the 10-year ASCVD risk score. As shown in Table 5, in men, the non-linear relationship between iron status and ASCVD risk score was consistent across hs-CRP strata. However, a significant interaction between hs-CRP and iron status in relation to ASCVD was observed in women (*P* interaction for SF = 0.004;* P* interaction for Hb = 0.003, Table 5). The associations of iron status with ASCVD risk were stronger for individuals with higher hs-CRP (> 1.3 mg/L) than those with lower hs-CRP (≤ 1.3 mg/L). In hypertensive women with lower hs-CRP, the adjusted *β* (95% CIs) from the lowest to the highest tertile of SF were 0.0, 1.50 (0.77, 2.23) and 1.87 (1.12, 2.62), and of Hb were 0.0, − 0.02 (− 0.78, 0.74) and − 0.20 (− 0.99, 0.58). In hypertensive women with higher hs-CRP, the adjusted *β* (95% CIs) from the lowest to the highest tertile of SF were 0.0, 1.48 (0.52, 2.43) and 3.41 (2.16, 4.36), and of Hb were 0.0, 0.93 (− 0.11, 1.98) and 1.60 (0.63, 2.58).

**Table 5:** Association between iron status and 10-year ASCVD risk score stratified by hs-CRP levels (Adjusted for marital status, education level, current drinker, physical activity and BMI)

	Tertiles of SF (ng/mL)					Tertiles of Hb (g/L)				
	T1	T2	T3	*P* for trend^a^	*P*-interaction	T1	T2	T3	*P* for trend^a^	*P*-interaction
Men					0.280					0.585
hs-CRP ≤ 1.3 mg/L hs-CRP > 1.3 mg/L	0.0 (Reference)	− 1.03 (− 1.92, − 0.15)^*^	0.04 (− 0.87, 0.95)	0.646		0.0 (Reference)	− 1.01 (− 1.94, − 0.08)^*^	− 0.70 (− 1.62, 0.22)	0.176	
	0.0 (Reference)	− 0.96 (− 1.94, 0.02)	− 0.55 (− 1.52, 0.42)	0.436		0.0 (Reference)	− 0.55 (− 1.52, 0.41)	− 0.85 (− 1.90, 0.19)	0.110	
Women					0.004					0.003
hs-CRP ≤ 1.3 mg/L	0.0 (Reference)	1.50 (0.77, 2.23)^***^	1.87 (1.12, 2.62)^***^	< 0.001		0.0 (Reference)	− 0.02 (− 0.78, 0.74)	− 0.20 (− 0.99, 0.58)	0.608	
hs-CRP > 1.3 mg/L	0.0 (Reference)	1.48 (0.52, 2.43)^**^	3.41 (2.16, 4.36)^***^	< 0.001		0.0 (Reference)	0.93 (− 0.11, 1.98)	1.60 (0.63, 2.58)^**^	0.002	

*ASCVD*, atherosclerotic cardiovascular disease; *Hb*, haemoglobin; *hs****-****CRP*, high sensitivity C-reactive protein; *SF*, serum ferritin; *T*, tertile

^a^*P* for trend were performed by assigning each participant the median value of including the tertile of iron status and treating it as a continuous variable

^*^P < 0.05; ^**^P < 0.01; ^***^P < 0.001

## Discussion

In this study that was performed on hypertensive patients, we found that iron status is strongly associated with the predicted 10-year ASCVD risk score and that the dose–effect curve varies by sex. Men showed a U-shaped curve, whereas women showed a linear positive correlation. Moreover, a significant interaction between iron status and hs-CRP in relation to the predicted ASCVD risk was observed in women.

Since the publication of the first study on the relationship between iron status and cardiovascular disease in 1981 [5], a rising number of epidemiological studies have discovered a link between high iron status and increased risk of ASCVD [6, 7, 14, 29]. There are also experimental studies reporting the relationship between iron and the cardiovascular system, but they are not in accordance, e.g. iron decreased contractility in isolated rat cardiomyocytes [30] but there was not a relationship between contractility and iron in the heart in a whole animal model [31]. The Bruneck study, throughout a 5-year follow-up period, found a positive correlation between SF levels and ultrasound measures of carotid atherosclerosis progression [6]. Subsequently, Hb levels ≥ 170.0 g/L was reported to be positively associated with increased risk for cardiac events in a cohort of US veterans [14]. Furthermore, the Mendelian randomization technique provided evidence that higher iron status, manifested as elevated SF concentration and transferrin saturation, is associated with increased stroke risk [29]. Notably, these studies primarily focused on the effects of iron on the risk of cardiovascular disease in the general population [6, 7]. The existence of hypertension may perturb the iron-ASCVD association. Kim et al. recently indicated that the effect of Hb concentration on major adverse cardiovascular events was more pronounced in hypertensive patients, suggesting the need for in-depth studies in hypertensive populations [32]. Atherosclerosis is the main underlying pathology of ASCVD. According to Ross and Glomset’s “Response to Injury” theory, atherosclerosis starts with endothelial cell injury [33]. It is now known that hypertension causes damage to the vascular endothelium [34]. On this basis, increased iron stores may further promote plaque formation through altered redox-sensitive signalling and inflammatory immune responses [35–37]. Some evidence suggested that iron overload can promote foam cell infiltration and necrotic core expansion, and thereby exacerbates the severity of atherosclerosis [38]. Furthermore, high Hb concentration can raise blood viscosity, which in turn increases peripheral resistance and decreases blood flow and perfusion [39]. Collectively, high iron may act synergistically with hypertension to promote atherosclerosis. The current study expands the body of knowledge by demonstrating a strong association between high iron status and increased ASCVD risk, highlighting the potential harms of high iron in hypertensive patients.

At the other end of the spectrum, ID may also have a detrimental effect on the cardiovascular outcome. The Atherosclerosis Risk in Communities study showed that individuals with ferritin levels below 30 ng/mL were at higher risk of heart failure compared to those with normal ferritin levels [40]. The results from the Ludwigshafen Risk and Cardiovascular Health Study showed an association between low iron status and cardiovascular as well as total mortality with an approximately 10-year follow-up [41]. Clinically, ID is more often considered to be an independent risk factor for poor cardiovascular prognosis. In the field of ASCVD, low iron status has not received equal attention as iron overload. However, a recent study found that ID-induced upregulation of transferrin levels and iron-binding ability can induce hypercoagulability by deactivating antithrombin through the thrombin/Factor XIIa complex [42]. At the same time, ID-induced thrombocytosis can increase the propensity for thrombosis [43]. Considering that ID affects the production of various antioxidant enzymes such as catalase and cytochrome oxidase, ID can also lead to oxidative damage and endothelial dysfunction [44]. Taken together, ID can be an etiological factor for ASCVD. In particular, Chang et al. identified a distinct correlation between prior ID and ischemic stroke in a case–control study comprising 204,372 individuals [45]. Contrary to the overwhelming evidence that anaemia and ID poses a significant risk to cardiovascular prognosis in patients with heart failure [46, 47], the clinical significance in hypertensive patients remains unclear. Only one study has pointed to anaemia as an important risk factor for cardiovascular and renal events in hypertensive patients [48]. This study adds to the literature evidence on the correlation between low iron status and ASCVD risk in hypertensive men.

Notably, despite the levels of iron were significantly lower in women than in men, we discovered that low iron status was related to an increased risk of ASCVD in men but not in women. Consistent with our study, the previous literature indicated that men with low iron status have a stronger association with ischaemic heart disease, stroke and a short lifespan than women with low iron status [49–51]. The possible explanation for the sex difference is numerous and requires further investigation. First, it is plausible that the repeated low iron status caused by heavy and continuous blood loss during menstruation may make the cardiovascular system of women more tolerant and resistant [52]. Second, the age-specific distribution of ID and anaemia probably has a major role in the sex difference associated with ASCVD risk. Men with anaemia and ID were predominantly older patients, while women were mainly younger subjects (Fig. S4). Therefore, younger age may be a factor explaining why low iron status is not associated with ASCVD risk score in female patients.

Inflammation affects iron regulation and is a recognised confounding factor [27]; we performed an interaction analysis of the iron status and inflammation. As a result, a stronger positive association between iron status and ASCVD risk was found in women with above-median hs-CRP relative to those with low hs-CRP. In response, we propose two biologically plausible explanations. The first explanation is that, as an acute-phase protein, the synthesis of ferritin is affected by inflammation and oxidative stress [53]. A review article has indicated that inflammatory cytokines, including the tumour necrosis factor α, interleukin 1α and interleukin 1β, can upregulate ferritin expression [54]. The second explanation is that inflammation and oxidative stress affect the normal structure and function of erythrocytes. As noted in the literature, inflammation and oxidative stress induce ferri- and ferryl Hb production [55, 56] and the subsequent release of heme and iron triggers a chain reaction to the vascular system that is toxic and leads to the development of atherosclerosis [57, 58]. The reason why the interaction was not found in men is unclear and still needs to be explored further.

This study is one of the few to explore the iron status and the predicted ASCVD risk in a hypertensive population. Hypertension may synergise with a disturbed iron status to promote the development of ASCVD. Therefore, the optimal range of iron under hypertension remains to be been determined. However, it should be admitted that there are several limitations. Firstly, we cannot establish the causal relation between iron status and the ASCVD risk, owing to the cross-sectional design. Secondly, the results were derived from Chinese hypertensive patients, which limits its applicability to other populations. Thirdly, although we excluded chronic diseases that may affect the iron status, and adjusted for several potential confounding factors, the unmeasured or incompletely measured factors may remain. Finally, we define SF less than 30 ng/mL as ID. Although prior research has confirmed its excellent positive predictive value for iron deficiency [59], chronic diseases and inflammation weaken its strength [26, 60]. Within our analysis, we included Hb as a way to mitigate the potential limitation. Nevertheless, further consideration of other iron parameters in the subsequent studies will be required to adequately resolve this concern.

In conclusion, in the hypertensive patients, there was a sex-specific association between iron status and predicted 10-year ASCVD risk. A U-shape curve is suggested in hypertensive men, in which ASCVD risk is increased in the extreme lower and higher iron status. While for hypertensive women, ASCVD risk increased with increasing iron levels. Further work is required to confirm the effects of sex on the iron-ASCVD association, in particular, to elucidate the potential reasons for these findings.

## Supplementary Information

Below is the link to the electronic supplementary material.

ESM 1 (DOCX 26 kb)

ESM 2 (DOCX 28 kb)

ESM 3 Fig. S1 Flow chart showing the derivation of the current study population. (PNG 1837 kb)

High resolution image (TIF 5251 kb)

ESM 4 Fig. S2 Frequency of log-transformed SF and Hb concentrations. Hb: haemoglobin; SF, serum ferritin. (PNG 617 kb)

High resolution image (TIF 29381 kb)

ESM 5 Fig. S3 Relation between log-transformed SF and Hb. Hb: haemoglobin; SF, serum ferritin. (PNG 416 kb)

High resolution image (TIF 2143 kb)

ESM 6 Fig. S4 Prevalence of ID and anaemia in different age groups. ID, iron deficiency. (PNG 124 kb)

High resolution image (TIF 17974 kb)

## Data Availability

The datasets generated and analysed during the current study are available from the corresponding author upon reasonable request.
