# Supplementary material for: Sex-Specific Association Between Iron Status and the Predicted 10-Year Risk for Atherosclerotic Cardiovascular Disease in Hypertensive Patients
Source: Biol Trace Elem Res. 2022 Jan 24;200(11):4594–607. doi: 10.1007/s12011-021-03060-y (PMC9492579; doi:10.1007/s12011-021-03060-y)
Supplement: Supplementary file 1 — (DOCX 26 kb) [file 12011_2021_3060_MOESM1_ESM.docx]

**Supplementary Materials**

**Sex-specific association between iron status and the predicted 10-year risk for atherosclerotic cardiovascular disease in hypertensive patients**

Juan Zhou ^1,2^ • Rui Zhao ^2^ • Dongxia Wang ^2^ • Qin Gao ^3^ • Dan Zhao ^1^ • Binfa Ouyang ^1^ • Liping Hao ^2*^• Xiaolin Peng ^1*^

^1^ Shenzhen Nanshan Center for Chronic Disease Control, Shenzhen 518051, China.

^2^ Department of Nutrition and Food Hygiene, Hubei Key Laboratory of Food Nutrition and Safety, and Ministry of Education Key Laboratory of Environment and Health, School of Public Health, Tongji Medical College, Huazhong University of Science and Technology, Wuhan 430030, China.

^3^ Department of Public Health, Jining Medical University, Jining 272067, China.

**Corresponding author:** Xiaolin Peng, Department of Oncology, Injury Prevention and Nutrition, Shenzhen Nanshan Center for Chronic Disease Control, 7 Huaming Road, Shenzhen 518051, China. Tel.: +86 755 26404209. Fax: +86 755 26403300. E-mail: xiaolinpeng@hotmail.com.

^*^ Xiaolin Peng and Liping Hao contributed equally to this paper.

Number of Supplementary Tables: 2

**Table S1** Characteristics of the hypertensive population according to tertiles of iron status in men (n=718) ^a^

| **Components of China-PAR model** | **Tertiles of SF** | | | | **Tertiles of Hb** | | | |
| --- | --- | --- | --- | --- | --- | --- | --- | --- |
|  | **T1** | **T2** | **T3** | ***P* for trend** ^b^ | **T1** | **T2** | **T3** | ***P* for trend** ^b^ |
| **Geographic region, n (%)** |  |  |  | - |  |  |  | - |
| Northern | 237 (100.0) | 244 (100.0) | 237 (100.0) |  | 221 (100.0) | 260 (100.0) | 237 (100.0) |  |
| Southern | 0 (0.0) | 0 (0.0) | 0 (0.0) |  | 0 (0.0) | 0 (0.0) | 0 (0.0) |  |
| **Urbanization, n (%)** |  |  |  | - |  |  |  | - |
| Urban | 237 (100.0) | 244 (100.0) | 237 (100.0) |  | 221 (100.0) | 260 (100.0) | 237 (100.0) |  |
| Rural | 0 (0.0) | 0 (0.0) | 0 (0.0) |  | 0 (0.0) | 0 (0.0) | 0 (0.0) |  |
| **Current smoker, n (%)** |  |  |  | 0.356 |  |  |  | 0.001 |
| No | 177 (74.7) | 174 (71.3) | 168 (70.9) |  | 175 (79.2) | 189 (72.7) | 155 (65.4) |  |
| Yes | 60 (25.3) | 70 (28.7) | 69 (29.1) |  | 46 (20.8) | 71 (27.3) | 82 (34.6) |  |
| **Diabetes mellitus, n (%)** |  |  |  | 0.246 |  |  |  | 0.024 |
| No | 193 (81.4) | 202 (82.8) | 183 (77.2) |  | 166 (75.1) | 214 (82.3) | 198 (83.5) |  |
| Yes | 44 (18.6) | 42 (17.2) | 54 (22.8) |  | 55 (24.9) | 46 (17.7) | 39 (16.5) |  |
| **Family history of ASCVD, n (%)** |  |  |  | 0.488 |  |  |  | 0.987 |
| No | 194 (81.9) | 195 (79.9) | 188 (79.3) |  | 175 (79.2) | 214 (82.3) | 188 (79.3) |  |
| Yes | 43 (18.1) | 49 (20.1) | 49 (20.7) |  | 46 (20.8) | 46 (17.7) | 49 (20.7) |  |
| **Treatment of hypertension, n (%)** |  |  |  | 0.009 |  |  |  | 0.469 |
| No | 64 (27.0) | 85 (34.8) | 39 (16.5) |  | 56 (25.3) | 65 (25.0) | 67 (28.3) |  |
| Yes | 173 (73.0) | 159 (65.2) | 198 (83.5) |  | 165 (74.7) | 195 (75.0) | 170 (71.7) |  |
| **WC (cm)** | 90.58 ± 8.44 | 91.39 ± 9.70 | 91.38 ± 8.68 | 0.384 | 88.65 ± 9.05 | 92.20 ± 8.54 | 92.23 ± 8.90 | < 0.001 |
| **TC (mmol/L)** | 4.86 ± 0.97 | 4.93 ± 1.02 | 4.93 ± 1.04 | 0.500 | 4.70 ± 0.89 | 4.94 ± 1.08 | 5.06 ± 0.99 | < 0.001 |
| **HDL-C (mmol/L)** | 1.19 ± 0.24 | 1.15 ± 0.24 | 1.13 ± 0.23 | 0.003 | 1.17 ± 0.27 | 1.15 ± 0.22 | 1.15 ± 0.22 | 0.462 |
| **SBP (mmHg)** | 134.64 ± 12.55 | 132.77 ± 13.41 | 133.41 ± 13.93 | 0.420 | 132.11 ± 13.36 | 133.69 ± 13.12 | 134.88 ± 13.39 | 0.026 |
| **DBP (mmHg)** | 84.41 ± 10.06 | 85.55 ± 9.95 | 85.84 ± 10.64 | 0.157 | 81.91 ± 9.68 | 85.31 ± 9.72 | 88.36 ± 10.30 | < 0.001 |

ASCVD, atherosclerotic cardiovascular disease; DBP, diastolic blood pressure; Hb, haemoglobin; HDL-C, high-density lipoprotein cholesterol; SBP, systolic blood pressure; SF, serum ferritin; TC, total cholesterol; T, tertile; WC, waist circumference.

^a^ Data are expressed as the means ± SDs for normally distributed variables or counts (percentages) for categorical variables.

^b^ Linear trends were tested using linear regression for continuous variables (with the median levels of SF or Hb as a continuous variable included in the regression models), and χ2 with linear-by-linear association test for categorical variables.

**Table S2** Characteristics of the hypertensive population according to tertiles of iron status in women (n=708) ^a^

| **Components of China-PAR model** | **Tertiles of SF** | | | | **Tertiles of Hb** | | | |
| --- | --- | --- | --- | --- | --- | --- | --- | --- |
|  | **T1** | **T2** | **T3** | ***P* for trend** ^b^ | **T1** | **T2** | **T3** | ***P* for trend** ^b^ |
| **Geographic region, n (%)** |  |  |  |  |  |  |  |  |
| Northern | 234 (100.0) | 240 (100.0) | 234 (100.0) |  | 219 (100.0) | 228 (100.0) | 261 (100.0) |  |
| Southern | 0 (0.0) | 0 (0.0) | 0 (0.0) |  | 0 (0.0) | 0 (0.0) | 0 (0.0) |  |
| **Urbanization, n (%)** |  |  |  |  |  |  |  |  |
| Urban | 234 (100.0) | 240 (100.0) | 234 (100.0) |  | 219 (100.0) | 228 (100.0) | 261 (100.0) |  |
| Rural | 0 (0.0) | 0 (0.0) | 0 (0.0) |  | 0 (0.0) | 0 (0.0) | 0 (0.0) |  |
| **Current smoker, n (%)** |  |  |  | 0.064 |  |  |  | 0.450 |
| No | 234 (100.0) | 239 (99.6) | 231 (98.7) |  | 217 (99.1) | 227 (99.6) | 260 (99.6) |  |
| Yes | 0 (0.0) | 1 (0.4) | 3 (1.3) |  | 2 (0.9) | 1 (0.4) | 1 (0.4) |  |
| **Diabetes mellitus, n (%)** |  |  |  | < 0.001 |  |  |  | 0.895 |
| No | 195 (83.3) | 199 (82.9) | 159 (67.9) |  | 168 (76.7) | 183 (80.3) | 202 (77.4) |  |
| Yes | 39 (16.7) | 41 (17.1) | 75 (32.1) |  | 51 (23.3) | 45 (19.7) | 59 (22.6) |  |
| **Family history of ASCVD, n (%)** |  |  |  | 0.740 |  |  |  | 0.627 |
| No | 182 (77.8) | 182 (75.8) | 185 (79.1) |  | 165 (75.3) | 182 (79.8) | 202 (77.4) |  |
| Yes | 52 (22.2) | 58 (24.2) | 49 (20.9) |  | 54 (24.7) | 46 (20.2) | 59 (22.6) |  |
| **Treatment of hypertension, n (%)** |  |  |  | 0.211 |  |  |  | < 0.001 |
| No | 71 (30.3) | 61 (25.4) | 59 (25.2) |  | 79 (36.1) | 60 (26.3) | 52 (19.9) |  |
| Yes | 163 ( 69.7) | 179 (74.6) | 175 (74.8) |  | 140 (63.9) | 168 (73.7) | 209 (80.1) |  |
| **WC (cm)** | 83.81 ± 8.65 | 83.94 ± 8.57 | 85.82 ± 8.61 | 0.007 | 83.28 ± 8.90 | 83.64 ± 7.81 | 86.33 ± 8.86 | < 0.001 |
| **TC (mmol/L)** | 4.96 ± 1.07 | 5.14 ± 0.98 | 5.23 ± 1.46 | 0.017 | 4.83 ± 0.99 | 5.04 ± 1.05 | 5.40 ± 1.38 | < 0.001 |
| **HDL-C (mmol/L)** | 1.33 ± 0.25 | 1.33 ± 0.28 | 1.30 ± 0.25 | 0.152 | 1.30 ± 0.29 | 1.33 ± 0.25 | 1.33 ± 0.25 | 0.233 |
| **SBP (mmHg)** | 135.07 ± 15.15 | 135.54 ± 14.23 | 134.21 ± 15.54 | 0.477 | 133.95 ± 14.63 | 134.55 ± 14.38 | 136.13 ± 15.70 | 0.105 |
| **DBP (mmHg)** | 83.48 ± 9.62 | 81.18 ± 8.84 | 79.71 ± 10.63 | < 0.001 | 80.21 ± 10.74 | 80.65 ± 9.05 | 83.19 ± 9.45 | < 0.001 |

ASCVD, atherosclerotic cardiovascular disease; DBP, diastolic blood pressure; Hb, haemoglobin; HDL-C, high-density lipoprotein cholesterol; SBP, systolic blood pressure; SF, serum ferritin; TC, total cholesterol; T, tertile; WC, waist circumference.

^a^ Data are expressed as the means ± SDs for normally distributed variables or counts (percentages) for categorical variables.

^b^ Linear trends were tested using linear regression for continuous variables (with the median levels of SF or Hb as a continuous variable included in the regression models), and χ2 with linear-by-linear association test for categorical variables.
