# Supplementary figures and images for: Sex-Specific Association Between Iron Status and the Predicted 10-Year Risk for Atherosclerotic Cardiovascular Disease in Hypertensive Patients
Source: Biol Trace Elem Res. 2022 Jan 24;200(11):4594–607. doi: 10.1007/s12011-021-03060-y (PMC9492579; doi:10.1007/s12011-021-03060-y)

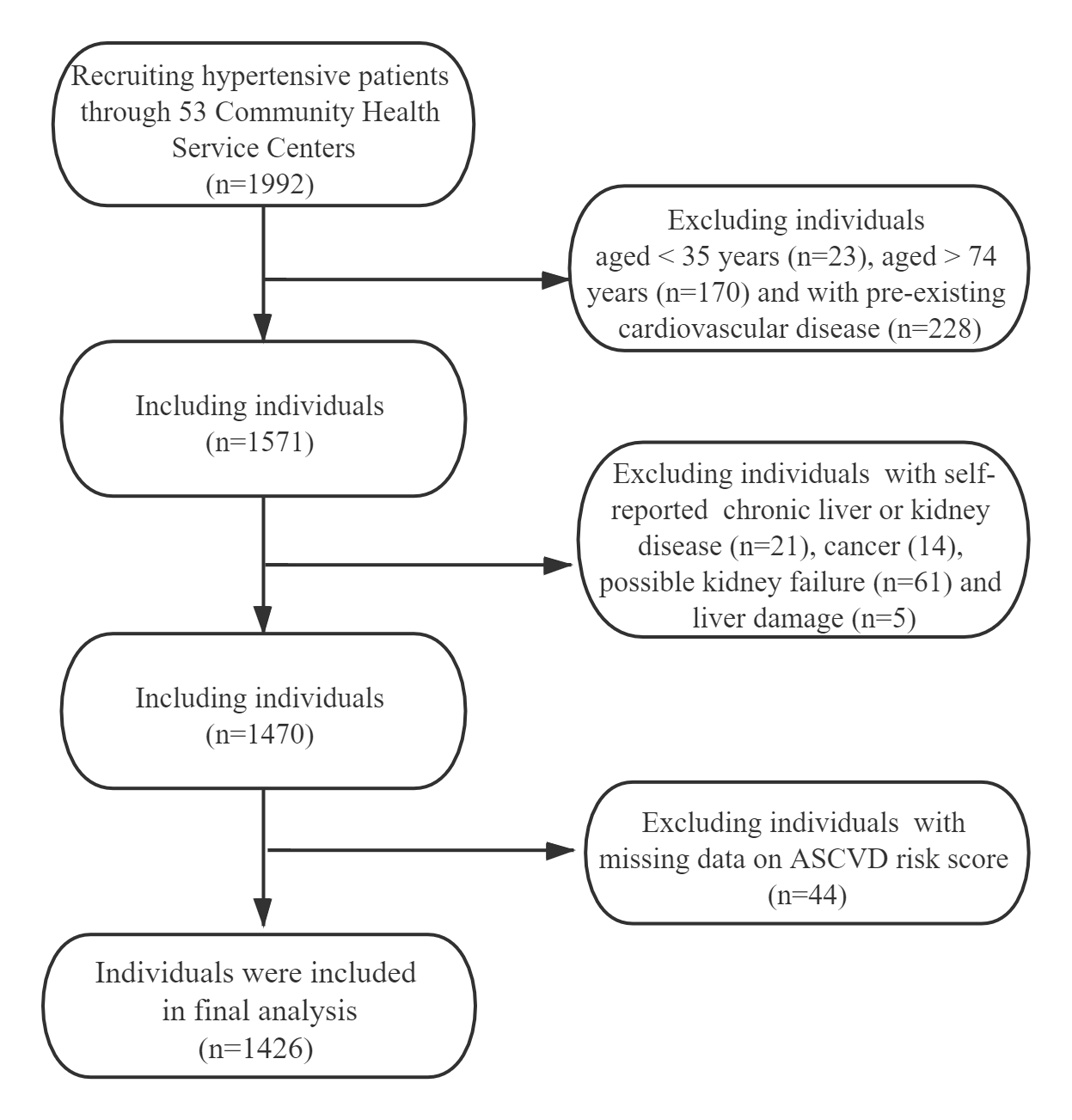

Supplement: Supplementary file 3 — Fig. S1 Flow chart showing the derivation of the current study population. (PNG 1837 kb) [file 12011_2021_3060_Fig3_ESM.png]

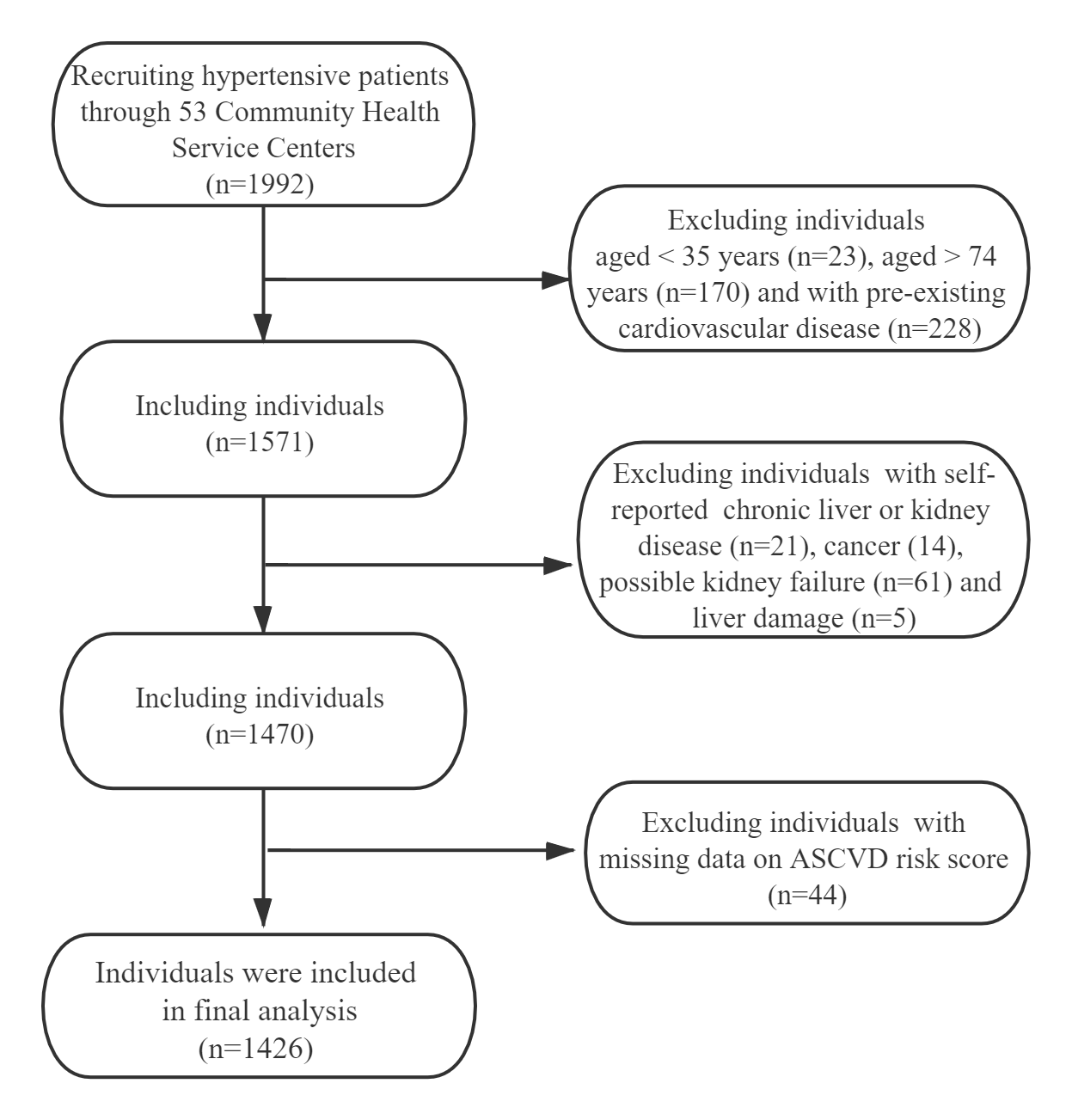

Supplement: Supplementary file 4 — High resolution image (TIF 5251 kb) [file 12011_2021_3060_MOESM3_ESM.tif]

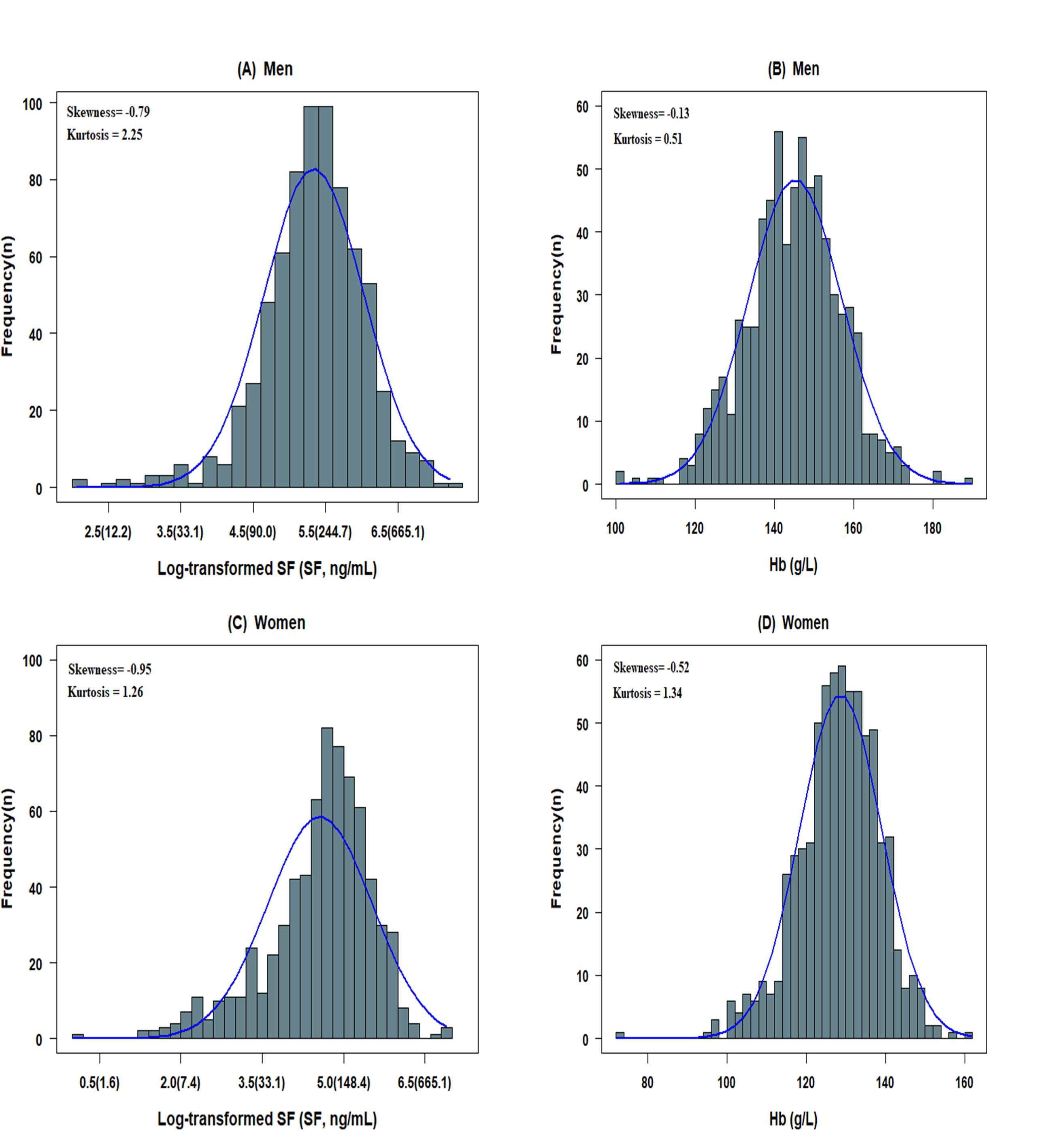

Supplement: Supplementary file 5 — Fig. S2 Frequency of log-transformed SF and Hb concentrations. Hb: haemoglobin; SF, serum ferritin. (PNG 617 kb) [file 12011_2021_3060_Fig4_ESM.png]

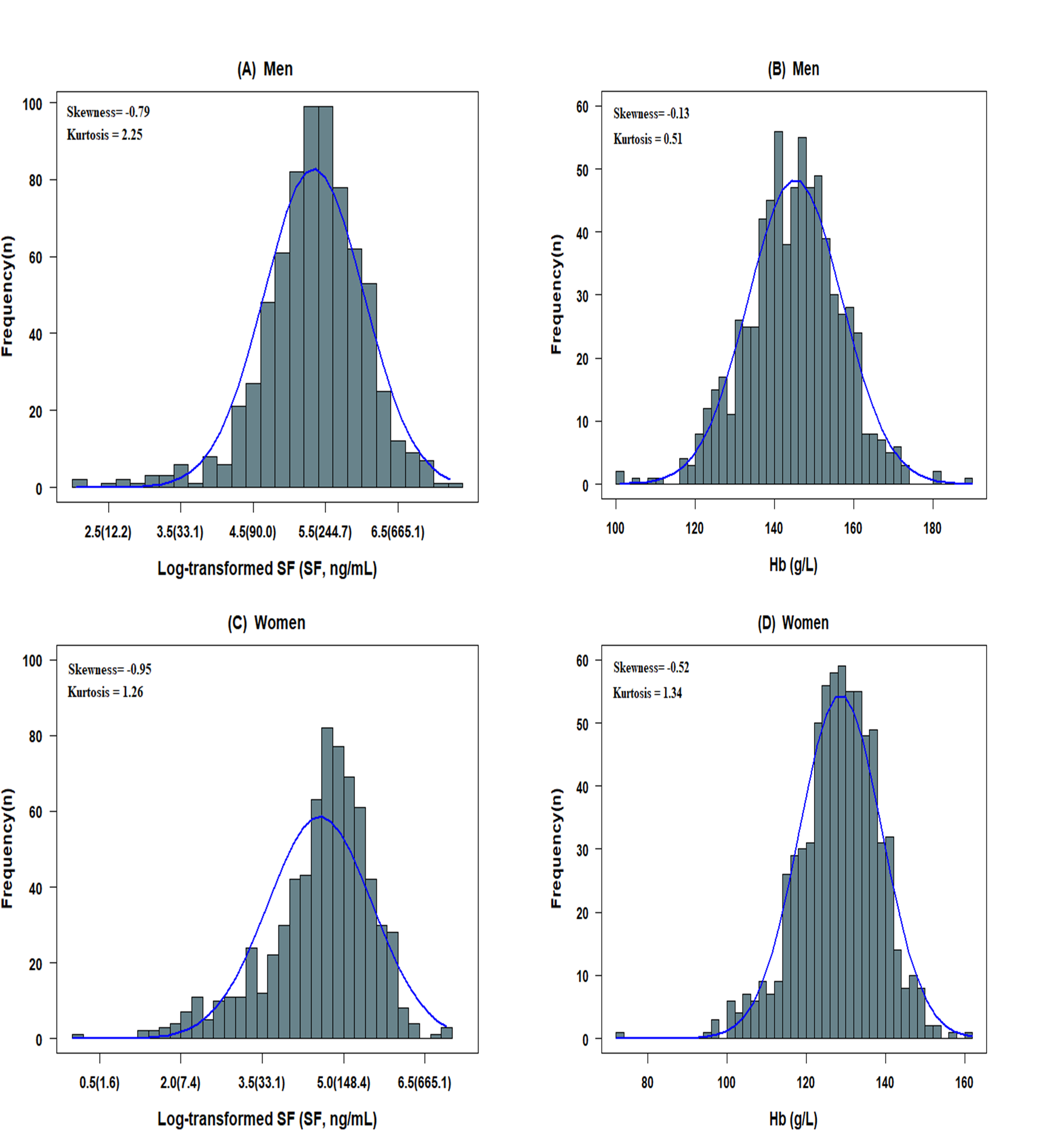

Supplement: Supplementary file 6 — High resolution image (TIF 29381 kb) [file 12011_2021_3060_MOESM4_ESM.tif]

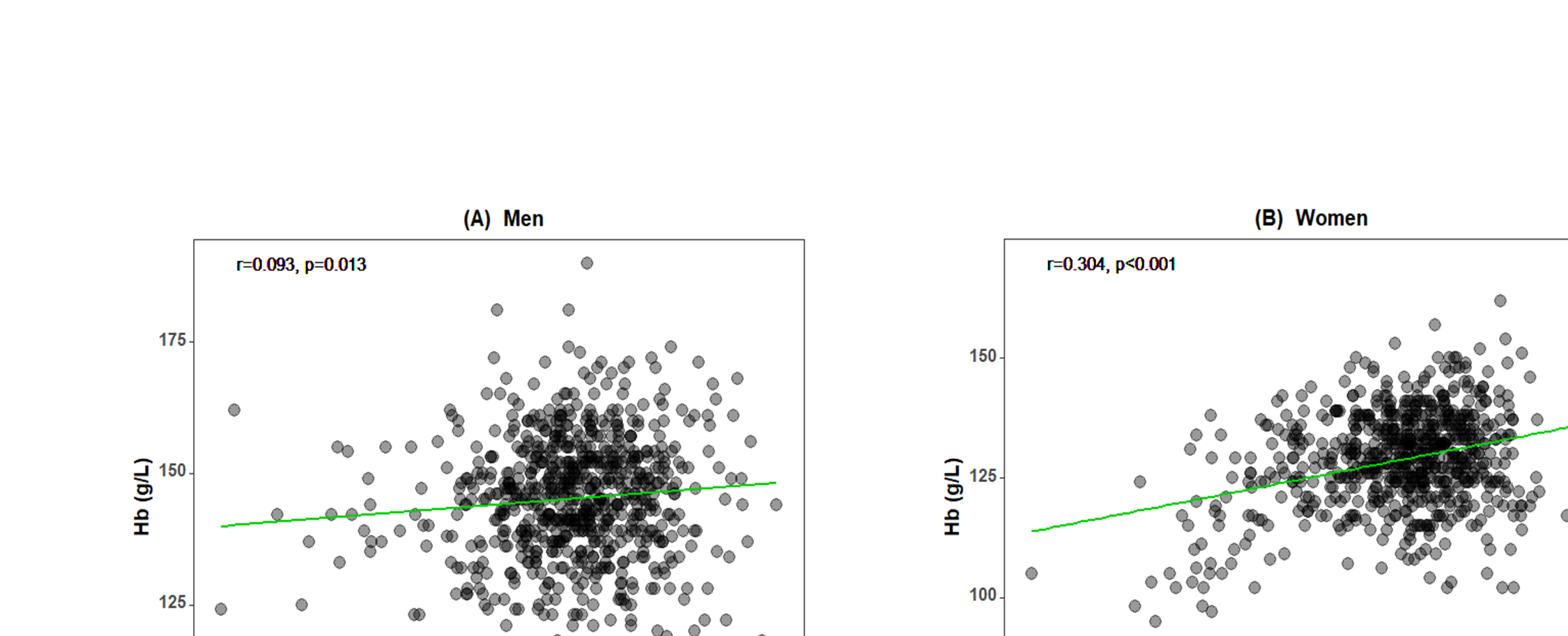

Supplement: Supplementary file 7 — Fig. S3 Relation between log-transformed SF and Hb. Hb: haemoglobin; SF, serum ferritin. (PNG 416 kb) [file 12011_2021_3060_Fig5_ESM.png]

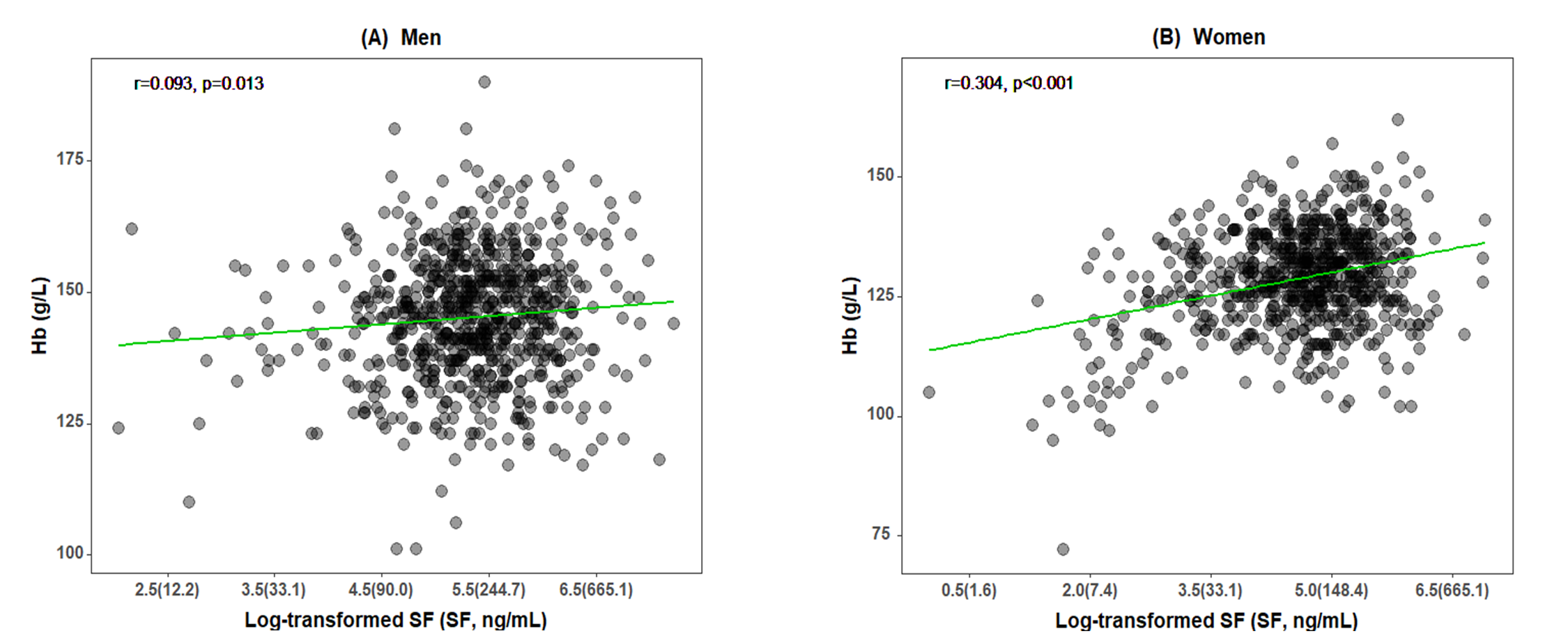

Supplement: Supplementary file 8 — High resolution image (TIF 2143 kb) [file 12011_2021_3060_MOESM5_ESM.tif]

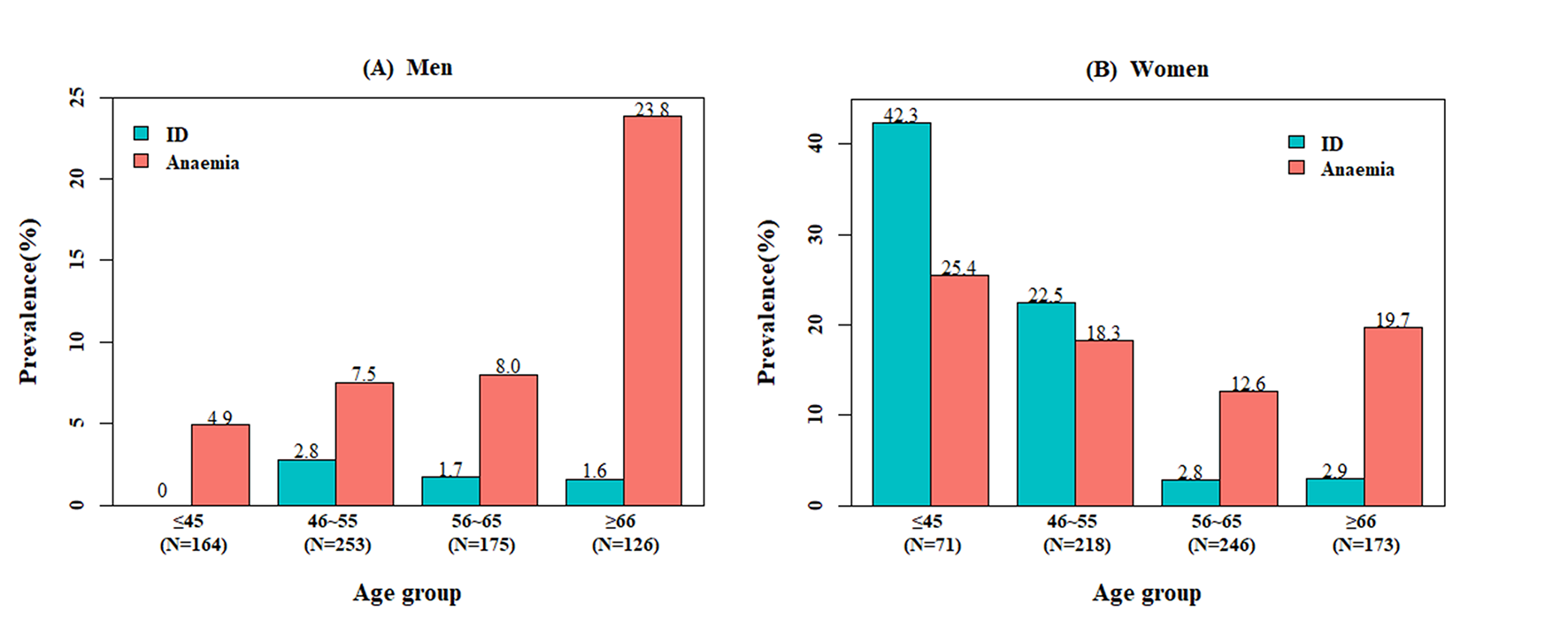

Supplement: Supplementary file 9 — Fig. S4 Prevalence of ID and anaemia in different age groups. ID, iron deficiency. (PNG 124 kb) [file 12011_2021_3060_Fig6_ESM.png]

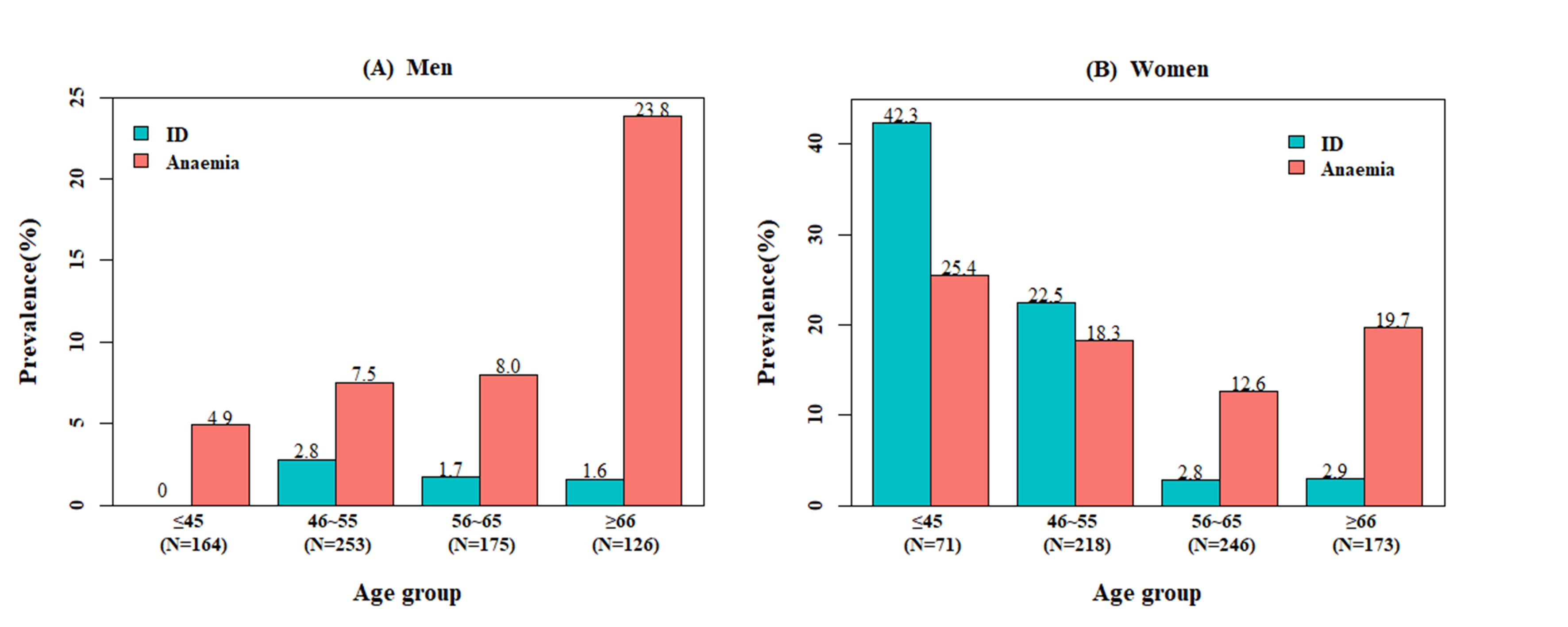

Supplement: Supplementary file 10 — High resolution image (TIF 17974 kb) [file 12011_2021_3060_MOESM6_ESM.tif]
